# Health-Related Benefits of Attaining the 8-Hr Ozone Standard

**DOI:** 10.1289/ehp.7186

**Published:** 2004-10-07

**Authors:** Bryan J. Hubbell, Aaron Hallberg, Donald R. McCubbin, Ellen Post

**Affiliations:** ^1^U.S. Environmental Protection Agency, Office of Air Quality Planning and Standards, Innovative Strategies and Economics Group, Research Triangle Park, North Carolina, USA; ^2^Abt Associates, Inc., Bethesda, Maryland, USA

**Keywords:** air pollution, benefit analysis, health impact assessment, ozone, standards

## Abstract

During the 2000–2002 time period, between 36 and 56% of ozone monitors each year in the United States failed to meet the current ozone standard of 80 ppb for the fourth highest maximum 8-hr ozone concentration. We estimated the health benefits of attaining the ozone standard at these monitors using the U.S. Environmental Protection Agency’s Environmental Benefits Mapping and Analysis Program. We used health impact functions based on published epidemiologic studies, and valuation functions derived from the economics literature. The estimated health benefits for 2000 and 2001 are similar in magnitude, whereas the results for 2002 are roughly twice that of each of the prior 2 years. The simple average of health impacts across the 3 years includes reductions of 800 premature deaths, 4,500 hospital and emergency department admissions, 900,000 school absences, and > 1 million minor restricted activity days. The simple average of benefits (including premature mortality) across the 3 years is $5.7 billion [90% confidence interval (CI), 0.6–15.0] for the quadratic rollback simulation method and $4.9 billion (90% CI, 0.5–14.0) for the proportional rollback simulation method. Results are sensitive to the form of the standard and to assumptions about background ozone levels. If the form of the standard is based on the first highest maximum 8-hr concentration, impacts are increased by a factor of 2–3. Increasing the assumed hourly background from zero to 40 ppb reduced impacts by 30 and 60% for the proportional and quadratic attainment simulation methods, respectively.

The Clean Air Act [U.S. Environmental Protection Agency (EPA) 1970] identified tropospheric ozone as one of six “criteria pollutants”—pervasive pollutants considered harmful to human health. Tropospheric ozone forms as a result of atmospheric reactions of nitrogen oxides (NO*_x_*) and volatile organic compounds (VOCs) in the presence of sunlight. Both local emissions sources, such as traffic, and emissions transported from upwind sources, such as electric utilities, contribute to ambient ozone levels in populated areas.

In 1997, the U.S. EPA changed the ozone standard to 80 ppb to reflect new scientific studies showing that ozone causes health effects at levels lower than the previous 120 ppb standard. Additionally, the form of the standard was changed to reflect studies showing that exposure times longer than 1 hr are of concern. The U.S. EPA set the form of the standard, which is the threshold for compliance and violations, at the fourth highest daily maximum 8-hr average occurring each year, averaged over a 3-year period.

New scientific studies published since 1996 have increased the body of evidence supporting the association between ambient ozone and a number of serious health effects (Anderson et al. 2004). For example, studies examining the association between ambient ozone and premature mortality have increased the weight of evidence supporting this important health impact (Anderson et al. 2004; Thurston and Ito 2001).

Our purpose for this analysis was to assess the human health benefits of attaining the 8-hr ozone standard. We applied a damage function approach similar to those used in several recent U.S. EPA regulatory impact analyses, including those for the proposed Clean Air Interstate Rule and the final Clean Air Nonroad Diesel Rule (U.S. EPA 2004a, 2004b). We focused the assessment on the benefits that might have been achieved if current monitored ozone levels (represented by the years 2000–2002) were reduced just to the levels required to meet the 8-hr standard. We conducted analyses to examine the sensitivity of our results to a number of different assumptions about the form of the standard, background levels of ozone, methods for simulating attainment of the 8-hr ozone standard, and the choice of health effects and effect estimates from published epidemiologic studies.

In this article, we provide detailed descriptions of the data and methods in this analysis, along with the results. We describe monitored ozone levels in 2000, 2001, and 2002, provide details on how we assigned monitored ozone levels to populations to estimate population-level exposures, and outline the two approaches we used to simulate attainment. We then discuss the literature on ozone-related health effects, describe the specific set of health impact functions we used in the benefits analysis and the economic values selected to estimate the dollar value of ozone-related health impacts, and discuss how we addressed uncertainty in the analysis. Finally, we present the results and implications of the analysis.

## Simulation of Changes in Population-Level Exposures to Ambient Ozone Due to Attainment

### Selecting monitoring data.

To estimate population-level ozone concentrations, we began by obtaining ozone monitoring data from the U.S. EPA’s Air Quality System (AQS) (U.S. EPA 2004c), a database of ambient air pollution data collected by the U.S. EPA, state, local, and tribal air pollution control agencies from > 1,000 monitoring stations across the country. We analyzed these data using the Environmental Benefits Mapping and Analysis Program (BenMAP), developed by the U.S. EPA for use in estimating the health impacts and economic benefits associated with changes in ambient air pollution (you may obtain a copy of BenMAP by e-mailing a request to B.J.H.). We used SAS (release 8.02; SAS Institute Inc., Cary, NC) to process the AQS data for use in BenMAP.

To characterize ozone levels, we selected monitors following criteria generally consistent with those the U.S. EPA uses to determine attainment and nonattainment of the 8-hr standard. We selected monitors that had a sufficient number of observations during the ozone “season,” which stretches from 1 May through 30 September, 153 days. Many areas of the United States, including Southern California and Texas, have a longer ozone season. Accounting for the longer ozone season in these areas would lead to an increase in the estimated benefits of attaining the standards.

Because missing monitor observations are common, we selected only those monitors that had observations on at least half the days in this period. Specifically, each monitor had to have at least 77 valid days, with a valid day defined as having at least nine hourly observations between 0800 and 1950 hr. We did not use data from any monitor with a parameter occurrence code (POC) > 4 to avoid errors that may be introduced by using nonstandard monitors. (POC codes are used to distinguish among multiple monitors at the same site that are measuring the same parameter. In general, a higher POC code is assigned to monitors that are not the primary ozone monitor.) For those locations with more than one ozone monitor, we selected the monitor with the lowest POC code (e.g., we chose POC 1 rather than 2), and dropped any others.

Table 1 summarizes the distribution of monitored fourth highest maximum daily 8-hr average ozone concentrations across the 3 study years. In all years, at least 35% of monitors failed to meet the level of the standard. However, there was some variability between years in the proportion of nonattainment monitors and in the amount by which monitors exceeded the standard. In 2000 and 2001, < 40% of monitors exceeded the standard, and ≤5% of the monitors exceeded 100 ppb. In 2002, 56% of monitors had ozone levels that exceeded the standard, and 14% had ozone levels > 100 ppb. Monitored ozone levels in 2002 were higher in part because of meteorologic conditions favorable for ozone formation and transport of ozone precursors (U.S. EPA 2003).

Ozone concentrations show spatial patterns, with certain areas of the United States, including California, having consistently high ozone values from 2000 through 2002. Other areas, such as the Southeast and Northeast, varied a great deal across those years. This may result from differences in climatic variability, natural phenomena such as wildfires, or differences in ozone precursor (NO*_x_* and VOC) emissions. Year-to-year precursor emissions may vary because of economic cycles; changes in electricity generation, such as switching from coal to natural gas; or changes in vehicle use.

### Applying spatial interpolation.

Monitor data represent ambient ozone levels at a series of discrete points in space. However, benefits analysis requires an estimate of ambient ozone concentrations for populations across the United States. For each year of monitoring data (2000, 2001, and 2002), we generated estimates of average ambient ozone levels for every county in the United States using applied spatial interpolation methods. Our base case analysis used Voronoi neighbor averaging (VNA), an algorithm that estimates ambient ozone levels by selecting the closest neighboring monitors surrounding the center of each county and then calculating the inverse distance weighted average of the monitor values for the selected neighboring monitors (e.g., Chen et al. 2004; Gold 1997). This method provides a relatively smooth surface in densely monitored areas.

We analyzed the accuracy of the VNA interpolation procedure by dropping individual monitors and predicting their ambient ozone levels using the remaining monitors. The national average differences between predicted and observed annual averages are < 1% in all cases, with standard deviations ranging from 10 to 12%. The largest differences occurred in rural areas and large portions of the western United States, where few monitors are present; ozone estimates in these cases are often based partially on monitors that are quite distant.

Most populations live within 50 km of an ozone monitor, however, so we can be reasonably confident that estimates of ambient ozone levels will be acceptable for most populated areas. We explored the sensitivity of the results to the choice of spatial interpolation method by estimating ambient ozone levels using a distance limited version of VNA (where all monitors farther than 50 km are discarded when choosing neighbors), as well as using a simple closest monitor assignment. A detailed explanation of each of these methods is provided in the Supplemental Material (http://ehp.niehs.nih.gov/docs/2004/7186/suppl.pdf).

### Reducing ozone levels to meet the standard.

To demonstrate the benefits of attaining the 8-hr standard in 2000, 2001, and 2002, we specified how ozone levels would be reduced to bring the specific attainment “metric” (fourth highest daily maximum 8-hr average) down to the level of the standard. The U.S. EPA’s primary (for health protection) and secondary (for environmental and welfare protection) 8-hr ozone standards both are 80 ppb. In determining attainment and nonattainment, however, the U.S. EPA must use rounding. As a result, we consider ozone values ≤84 ppb as meeting the standard.

There are several ways to reduce the distribution of hourly ozone values to simulate attainment. For simplicity we treated the form of the standard as simply the fourth highest daily maximum 8-hr average, rather than the fourth highest daily maximum 8-hr average averaged over the 3 previous years.

We investigated two different methods: percentage (or proportional) rollback and quadratic rollback. Percentage rollback simply reduces all daily metric values by the percentage required to bring the violating day (the day with the fourth highest value) down to 84 ppb. The quadratic rollback method reduces larger metric values proportionally more than smaller values.

It is not clear which method provides a more realistic simulation of an attainment strategy. If control strategies affect emissions on all days during the ozone season, then using percentage rollback may be appropriate. If control strategies affect emissions on days with higher ozone levels more than on days with lower levels, then quadratic rollback may be more realistic. Both of these approaches represent implementation strategies that areas may select to meet the ozone standard. See Supplemental Material (http://ehp.niehs.nih.gov/docs/2004/7186/suppl.pdf) for more details on the two methods.

For both methods, we assume a constant background 8-hr daily maximum ozone level of 40 ppb, representing the amount of ozone (for this averaging period) that is not attributable to U.S. anthropogenic sources. It is assumed that this background cannot be affected by attempts to attain the ozone standards, and thus this portion of the estimated ambient ozone levels is not adjusted by either rollback method.

Vingarzan (2004) surveyed recent literature on background ozone concentrations and concluded that based on data from 1983 to 2001, median background levels in the United States ranged between 13 and 47 ppb. Vingarzan (2004) notes that background levels appear to be increasing over time because of increased contributions from international transport of ozone precursors. Therefore, we selected a background ozone level toward the upper end of the observed range because our monitor data are based on later years. The background level likely varies across the United States, and our assumption of 40 ppb adds uncertainty to the analysis (Vingarzan 2004). We investigated the impact of different assumptions about background levels of the attainment metric in a sensitivity analysis.

Once BenMAP has calculated how the attainment metric will be affected for each day, it calculates how the other ozone metrics required for the various health impact functions will be affected. These include daily maxima for 1-hr and 8-hr periods, as well as daily averages over different time periods, including the 24-hr average, the 5-hr average (1000–1450 hours), and the 8-hr average (0900–1650 hours). To calculate, BenMAP rolls back individual hourly ozone observations such that they meet the target metric values. For details on this process, see Supplemental Material (http://ehp.niehs.nih.gov/docs/2004/7186/suppl.pdf).

In adjusting individual hourly ozone values to meet the target metric value, we assumed that there is no fixed background level of ozone for any particular hour and set the background to zero. Any given hourly value may have a specific background component; however, we are unable to determine what this component might be. We examined the impact of assuming alternative hourly background levels as a sensitivity analysis.

Finally, BenMAP uses the adjusted hourly values to calculate the adjusted ozone summary measures—for example, 24-hr average, 1-hr maximum, and the like. Using the three methods described above, BenMAP then spatially interpolates the set of adjusted summary measures to the center of each county. The differences between the spatially interpolated baseline and the adjusted summary measures are the basic air quality inputs to the health benefits model.

Note that BenMAP does not adjust monitors that meet the attainment test (those with fourth highest maximum daily 8-hr average ≤84 ppb). However, these monitors are included in the interpolation process, so the ozone levels assigned to a population in a given county will, in most cases, reflect an average of monitors with ozone reductions and those with no reduction. In reality, there will be reductions in ozone levels at monitors in a non-attainment area because of controls applied to meet the standard. Therefore, we are likely underestimating the change in ambient ozone that would occur as the result of implementing attainment strategies.

## Health Impact Functions

Health impact functions measure the change in a health end point of interest, such as hospital admissions, for a given change in ozone. Health impact functions are derived from the epidemiology literature. A standard health impact function has four components: an effect estimate from a particular epidemiologic study, a baseline incidence rate for the health effect (obtained from either the epidemiology study or a source of public health statistics, e.g., the Centers for Disease Control and Prevention), the affected population, and the estimated change in the relevant ozone summary measures.

A typical health impact function might be as follows:


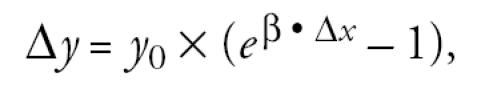


where *y*_0_ is the baseline incidence, equal to the baseline incidence rate times the potentially affected population; β is the effect estimate; and Δ*x* is the estimated change in the summary ozone measure. There are other functional forms, but the basic elements remain the same. The following subsections describe the sources for each of the elements other than the ozone air quality inputs to the health impact functions just described: affected populations, effect estimates, and baseline incidence rates.

### Affected Populations

The starting point for estimating affected populations is the 2000 U.S. Census block-level data set (Geolytics Inc. 2002). BenMAP incorporates 250 age/sex/race categories to match specific populations potentially affected by ozone and other air pollutants. The software constructs specific populations matching the populations in each epidemiologic study by accessing the appropriate age-specific populations from the overall population database. BenMAP projects populations to 2001 and 2002 using growth factors based on economic projections (Woods and Poole Economics Inc. 2001).

### Effect Estimate Sources

The most significant benefits of reducing ambient concentrations of ozone are attributable to reductions in health risks. The U.S. EPA’s Ozone Criteria Document (U.S. EPA 1996b) and the World Health Organization’s recent reports (Anderson et al. 2004) outline numerous health effects known or suspected to be linked to exposure to ambient ozone.

More than 1,000 new health and welfare studies have been published since the U.S. EPA issued the 8-hr ozone standard in 1997. Many of these studies investigated the impact of ozone exposure on health effects, such as changes in lung structure and biochemistry, lung inflammation, asthma exacerbation and causation, respiratory-illness–related school absence, hospital and emergency department (ED) visits for asthma and other respiratory causes, and premature death.

We excluded some health effects from this analysis for four reasons: the possibility of double counting (e.g., hospital admissions for specific respiratory diseases), uncertainties in applying effect relationships that are based on clinical studies to the affected population, a lack of an established concentration–response relationship, or the inability to appropriately value the effect (e.g., changes in forced expiratory volume) in economic terms. Table 2 lists the health end points included in the primary and sensitivity analyses for this article.

In selecting epidemiologic studies as sources of effect estimates, we applied several criteria to develop a set of studies that is likely to provide the best estimates of impacts in the United States. To account for the potential impacts of different health care systems or underlying health status of populations, we gave preference to U.S. studies over non-U.S. studies. In addition, because of the potential for confounding by copollutants, we gave preference to effect estimates from models including both ozone and particulate matter over single-pollutant models.

A number of end points that are not health related also may significantly contribute to monetized benefits. These include decreased outdoor worker productivity, decreased yields for commercial and noncommercial crops, decreased commercial forest productivity, damage to urban ornamental plants, impacts on recreational demand from damaged forest aesthetics, and damage to ecosystem functions (U.S. EPA 1996a, 1999). Estimation of these impacts is beyond the scope of this analysis.

#### Effect estimates: premature mortality.

Although particulate matter is the air pollutant most clearly associated with premature mortality, recent research suggests that repeated ozone exposure likely contributes to premature death. Several recent analyses have found consistent statistical associations between ozone exposure and increased mortality (Fairley et al. 2003; Toulomi et al. 1997). In addition, Bell et al. (2004) found an overall significant impact of ozone on mortality using an extended version of the National Morbidity, Mortality, and Air Pollution Study database. Their results were significant even after controlling for PM levels.

Although they do not constitute a database as extensive as that for particulate matter, these recent studies provide supporting evidence for including mortality in ozone health benefits analyses. Thurston and Ito (2001) reviewed previously published time-series studies examining the effect of daily ozone levels on daily mortality. They hypothesized that much of the variability in published estimates of the ozone–mortality effect could be explained by how well each model controlled for the influence of weather, an important confounder, and that earlier studies, which used less sophisticated approaches to controlling for weather, consistently underpredicted the ozone–mortality effect.

Thurston and Ito (2001) also found that models incorporating a nonlinear temperature specification appropriate for the U-shaped nature of the temperature–mortality relationship (i.e., increased deaths at both very low and very high temperatures) produced ozone–mortality effect estimates that were both more strongly positive (a 2% increase in relative risk over the pooled estimate for all studies evaluated) and consistently statistically significant. Further accounting for the interaction effects between temperature and relative humidity strengthened the positive effect. Including a particulate matter index to control for particulate matter (PM)–mortality effects had little effect on these results, suggesting a relationship between ozone and mortality independent of that for PM. However, most of the studies that Thurston and Ito (2001) examined controlled only for PM ≤10 μm (PM_10_) or broader measures of particles and did not directly control for PM ≤2.5 μm (PM_2.5_). Therefore, there still may be potential for confounding of PM_2.5_ and ozone mortality effects, given that ozone and PM_2.5_ are highly correlated during summer months in some areas.

Two recent World Health Organization reports found that “recent epidemiologic studies have strengthened the evidence that there are short-term O_3_ effects on mortality and respiratory morbidity and provided further information on exposure–response relationships and effect modification” (Anderson et al. 2004; WHO 2003). In addition, Levy et al. (2001) assessed the epidemiologic evidence regarding the link between short-term exposures to ozone and premature mortality. Based on four U.S. studies (Ito and Thurston 1996; Kelsall et al. 1997; Moolgavkar 2000; Moolgavkar et al. 1995a), they concluded that an appropriate pooled effect estimate is a 0.5% increase in premature deaths per 10 μg/m^3^ increase in 24-hr average ozone concentrations, with a 95% confidence interval (CI) between 0.3 and 0.7%.

We included ozone mortality in the base health effects estimate for the ozone benefits reanalysis, with the recognition that the exact magnitude of the effects estimate is subject to continuing uncertainty. We used results from three U.S. studies to calculate the base-case ozone mortality estimate. We selected these studies (Ito and Thurston 1996; Moolgavkar et al. 1995a; Samet et al. 1997) based on the logic that the demographic and environmental conditions existing when these studies were conducted would, on average, be most similar (relative to international studies) to the conditions prevailing when the ozone standards would be implemented. We examined the impact of including a fourth U.S. study by Kinney et al. (1995) in a sensitivity analysis. We excluded Kinney et al. (1995) from the primary analysis because, as Levy et al. (2001) noted, that study included only a linear term for temperature. Because Kinney et al. (1995) found no significant ozone effect, including this study in the primary analysis would lead to an underestimate of true mortality impacts and increase the uncertainty surrounding the estimated mortality reductions.

We then estimated the change in mortality incidence resulting from application of the effect estimate from each study and combined the results using a random-effects weighting procedure, discussed in the Supplemental Material (http://ehp.niehs.nih.gov/docs/2004/7186/suppl.pdf), that accounts for both the precision of the individual effect estimates and between-study variability. However, it is important to note that this procedure only captures the uncertainty in the underlying epidemiologic work and does not capture other sources of uncertainty, such as that in the estimation of air pollution exposure (Levy et al. 2001).

#### Effect estimates: respiratory hospital admissions.

Detailed hospital admission and discharge records provide data for an extensive body of literature examining the relationship between hospital admissions and air pollution. This is especially true for the population ≥65 years of age, because of the availability of detailed Medicare records. Because the number of hospital admission studies is so large, we used results from a number of studies to pool some hospital admission end points. In addition, there is one study (Burnett et al. 2001) providing an effect estimate for respiratory hospital admissions in children ≤2 years of age.

To estimate total respiratory hospital admissions associated with changes in ozone for adults ≥65 years of age, we first estimated the change in hospital admissions for the separate effect categories that each study provided for each city: Minneapolis, Minnesota; Detroit, Michigan; Tacoma, Washington; and New Haven, Connecticut. To estimate all respiratory hospital admissions for Detroit, we added the pneumonia and chronic obstructive pulmonary disease (COPD) estimates, based on the effect estimates given by Schwartz (1994b). Similarly, we summed the estimated hospital admissions based on the effect estimates that Moolgavkar et al. (1997) reported for Minneapolis. To estimate all respiratory hospital admissions for Minneapolis using Schwartz (1994a), we simply estimated pneumonia hospital admissions based on the effect estimate. Making this assumption that pneumonia admissions represent the total impact of ozone on hospital admissions will give some weight to the possibility that there is no relationship between ozone and COPD, reflecting the equivocal evidence represented by the different studies. We then used a fixed-effects pooling procedure to combine the two all-respiratory hospital-admission estimates for Minneapolis. Finally, we used random-effects pooling to combine the results for Minneapolis and Detroit, in addition to results for Tacoma and New Haven. As noted above, this pooling approach accounts for both the precision of the individual-effect estimates and the between-study variability characterizing differences across study locations.

#### Effect estimate: asthma-related ED visits.

We used three studies as the source for the concentration–response functions we used to estimate the effects of ozone exposure on asthma-related ED visits: Cody et al. (1992), Weisel et al. (1995), and Stieb et al. (1996). We estimated the change in ED visits using the effect estimate from each study and then pooled the results using the random-effects pooling procedure described in the Supplemental Material (http://ehp.niehs.nih.gov/docs/2004/7186/suppl.pdf). A more recent study by Jaffe et al. (2003) examined the relationship between ED visits and air pollution for people 5–34 years of age in the Ohio cities of Cleveland, Columbus, and Cincinnati from 1991 through 1996. We did not use this particular study in our primary analysis because it represents a more limited population and excludes potentially important impacts on the population ≥35 years of age. However, because many asthma-related ED visits involve children, this study was included in a sensitivity analysis showing the magnitude of results for all ages relative to those for a population more heavily weighted toward children. We included both hospital admissions and ED visits as separate end points associated with ozone exposure, because our estimates of hospital admission costs do not include the costs of ED visits.

#### Effect estimate sources: minor restricted activity days.

Minor restricted activity days (MRADs) occur when individuals reduce most usual daily activities and replace them with less strenuous activities or rest but do not miss work or school. We estimated the effect of ozone on MRADs using a concentration–response function derived from Ostro and Rothschild (1989). These researchers estimated the impact of ozone and PM_2.5_ on MRAD incidence in a national sample of the adult working population (18–65 years of age) living in metropolitan areas. We developed separate coefficients for each year of the Ostro and Rothschild (1989) analysis (1976–1981), which we then combined for use in the U.S. EPA’s analysis. The effect estimate used in the impact function is a weighted average of the coefficients in Ostro and Rothschild (1989, their Table 4), using the inverse of the variance as the weight.

#### Effect estimate: school absences.

Children may be absent from school because of respiratory or other acute diseases caused or aggravated by exposure to air pollution. Several studies have found a significant association between ozone levels and school absence rates. We use two recent studies (Chen et al. 2000; Gilliland et al. 2001) to estimate changes in school absences resulting from changes in ozone levels. Gilliland et al. (2001) estimated the incidence of new periods of absence, whereas Chen et al. (2000) examined absence on a given day. We converted the Gilliland et al. estimate to days of absence by multiplying the absence periods by the average duration of an absence. We estimated 1.6 days as the average duration of a school absence, the result of dividing the average daily school absence rate from Chen et al. (2000) and Ransom and Pope (1992) by the episodic absence rate from Gilliland et al. (2001). Thus, each Gilliland et al. period of absence is converted into 1.6 absence days.

Following recent advice from the National Research Council (2002), we calculated reductions in school absences for the full population of school-age children (5–17 years of age). This is consistent with recent peer-reviewed literature on estimating the impact of ozone on school absences (Hall et al. 2003). We estimated the change in school absences using both Chen et al. (2000) and Gilliland et al. (2001) and then pooled the results using the random-effects pooling procedure described in Supplemental Material (http://ehp.niehs.nih.gov/docs/2004/7186/suppl.pdf).

### Baseline Incidence Rates

Epidemiologic studies of the association between pollution levels and adverse health effects generally provide a direct estimate of the relationship of air quality changes to the relative risk of a health effect, rather than estimating the absolute number of avoided cases. For example, a typical result might be that a 100 ppb decrease in daily ozone levels might in turn decrease hospital admissions by 3%. The baseline incidence of the health effect is necessary to convert this relative change into a number of cases. A baseline incidence rate is the estimate of the number of cases of the health effect per year in the assessment location, because it corresponds to baseline pollutant levels in that location. To derive the total baseline incidence per year, this rate must be multiplied by the corresponding population number. For example, if the baseline incidence rate is the number of cases per year per 100,000 people, that number must be multiplied by the number of 100,000s in the population.

Table 3 summarizes the sources of baseline incidence rates and provides average incidence rates for the end points included in the analysis. For both baseline incidence and prevalence data, we used age-specific rates where available. We applied concentration–response functions to individual age groups and then summed over the relevant age range to provide an estimate of total population benefits. In most cases, we used a single national incidence rate, because of a lack of more spatially disaggregated data. Whenever possible, the rates used are national averages, because these data are most applicable to a national assessment of benefits. For some studies, however, the only available incidence information comes from the studies themselves; in these cases, incidence in the study population is assumed to represent typical incidence at the national level. Regional incidence rates are available for hospital admissions, and county-level data are available for premature mortality.

## Economic Values for Health Outcomes

Reductions in ambient concentrations of air pollution generally lower the risk of future adverse health effects for a large population. Therefore, the appropriate economic measure is willingness to pay (WTP) for changes in risk of a health effect rather than WTP for a health effect that would occur with certainty (Freeman 1993). Epidemiologic studies generally provide estimates of the relative risks of a particular health effect that is avoided because of a reduction in air pollution. We converted those to units of avoided statistical incidence for ease of presentation. We calculated the value of avoided statistical incidences by dividing individual WTP for a risk reduction by the related observed change in risk. For example, suppose a pollution-reduction regulation is able to reduce the risk of premature mortality from 2 in 10,000 to 1 in 10,000 (a reduction of 1 in 10,000). If individual WTP for this risk reduction is $100, then the WTP for an avoided statistical premature death is $1 million ($100/0.0001 change in risk).

WTP estimates generally are not available for some health effects, such as hospital admissions. In these cases, we used the cost of treating or mitigating the effect as a primary estimate. These cost-of-illness (COI) estimates generally understate the true value of reducing the risk of a health effect, because they reflect the direct expenditures related to treatment but not the value of avoided pain and suffering (Berger et al. 1987; Harrington and Portney 1987). We provide unit values for health end points (along with information on the distribution of the unit value) in Table 4. All values are in constant year 2000 US$, adjusted for growth in real income. Economic theory argues that WTP for most goods (e.g., environmental protection) will increase if real income increases. Many of the valuation studies used in this analysis were conducted in the late 1980s and early 1990s. Because real income has grown since the studies were conducted, people’s WTP for reductions in the risk of premature death and disease likely has grown as well. We did not adjust COI-based values, because they are based on current costs. Similarly, we did not adjust the value of school absences, because that value is based on current wage rates. Table 4 presents the values for individual end points adjusted to year 2000 income levels.

### Mortality.

To estimate the monetary benefit of reducing the risk of premature death, we used the “value of statistical lives” saved (VSL) approach, which is a summary measure for the value of small changes in mortality risk for a large number of people. The VSL approach applies information from several published value-of-life studies to determine a reasonable monetary value of preventing premature mortality. The mean value of avoiding one statistical death is estimated to be roughly $6 million in 2000 US$. This represents an intermediate value from a variety of estimates in the economics literature (U.S. EPA 1999).

### Respiratory hospital admissions.

In the absence of estimates of societal WTP to avoid hospital visits/admissions for specific illnesses, estimates of total COI (total medical costs plus the value of lost productivity) typically are used as conservative, or lower bound, estimates. These estimates are biased downward because they do not include the WTP value of avoiding pain and suffering.

The *International Classification of Diseases, 9th Revision* (ICD-9; International Classification of Diseases 1979) code-specific COI estimates we used in this analysis consist of estimated hospital charges and the estimated opportunity cost of time spent in the hospital (based on the average length of a hospital stay for the illness). We based all estimates of hospital charges and length of stays on statistics provided by the Agency for Healthcare Research and Quality (2000). We estimated the opportunity cost of a day spent in the hospital as the value of the lost daily wage, regardless of whether the hospitalized individual is in the workforce. To estimate the lost daily wage, we divided the 1990 median weekly wage by 5 and inflated the result to 2000 US$ using the urban consumer price index (CPI-U) (U.S. Bureau of Labor Statistics 2004) for “all items.” The resulting estimate is $109.35. The total COI estimate for an ICD code–specific hospital stay lasting *n* days, then, was the mean hospital charge plus $109 × *n*.

### Asthma-related ED visits.

To value asthma ED visits, we used a simple average of two estimates from the health economics literature. The first estimate comes from Smith et al. (1997), who reported approximately 1.2 million asthma-related ED visits in 1987, at a total cost of $186.5 million (1987 US$). The average cost per visit that year was $155; in 2000 US$, that cost was $311.55 (using the CPI-U for medical care to adjust to 2000 US$). The second estimate comes from Stanford et al. (1999), who reported the cost of an average asthma-related ED visit at $260.67, based on 1996–1997 data. A simple average of the two estimates yields a (rounded) unit value of $286.

### Minor restricted activity days.

No studies are reported to have estimated WTP to avoid an MRAD. However, Industrial Economics Inc. (unpublished data) has derived an estimate of WTP to avoid a minor respiratory restricted activity day, using estimates from Tolley et al. (1986) of WTP for avoiding a combination of coughing, throat congestion, and sinusitis. The IEc estimate of WTP to avoid a minor respiratory restricted activity day is $38.37 (1990 US$), or about $52 (2000 US$).

Although Ostro and Rothschild (1989) statistically linked ozone and MRADs, it is likely that most MRADs associated with ozone exposure are, in fact, minor respiratory restricted activity days. For the purpose of valuing this health end point, we used the estimate of mean WTP to avoid a minor respiratory restricted activity day.

### School absences.

To value a school absence, we *a*) estimated the probability that if a school child stays home from school, a parent will have to stay home from work to care for the child; and *b*) valued the lost productivity at the parent’s wage. To do this, we estimated the number of families with school-age children in which both parents work, and we valued a school-loss day as the probability that such a day also would result in a work-loss day. We calculated this value by multiplying the proportion of households with school-age children by a measure of lost wages.

We used this method in the absence of a preferable WTP method. However, this approach is likely to understate the value of school-loss days in three ways: First, it omits WTP to avoid the symptoms/illness that resulted in the school absence; second, it effectively gives zero value to school absences that do not result in work-loss days; and third, it uses conservative assumptions about the wages of the parent staying home with the child.

For this valuation approach, we assumed that in a household with two working parents, the female parent will stay home with a sick child. From the Statistical Abstract of the United States (U.S. Census Bureau 2001), we obtained *a*) the numbers of single, married, and “other” (widowed, divorced, or separated) working women with children; and *b*) the rates of participation in the workforce of single, married, and “other” women with children. From these two sets of statistics, we calculated a weighted average participation rate of 72.85%.

Our estimate of daily lost wage (wages lost if a mother must stay at home with a sick child) is based on the year 2000 median weekly wage among women ≥25 or more years of age (U.S. Census Bureau 2001). This median weekly wage is $551. Dividing by 5 gives an estimated median daily wage of $103. To estimate the expected lost wages on a day when a mother has to stay home with a school-age child, we first estimated the probability that the mother is in the workforce and then multiplied that estimate by the daily wage she would lose by missing a work day: 72.85% times $103, for a total loss of $75.

## Methods for Describing Uncertainty

Any complex analysis is likely to reflect many sources of uncertainty, and this analysis is no exception. We used numerous inputs to derive the benefits estimate, including measured ozone concentrations at monitor sites, interpolation methods, estimates of values (both from WTP and COI studies), population estimates, baseline incidence rate estimates, and income estimates. Each of these inputs may be uncertain, and depending on its location in the benefits analysis, each may have a disproportionately large impact on final estimates of total benefits. For example, we used measured ozone concentrations at monitor sites in the first stage of the analysis, meaning that any uncertainty in those measurements will propagate as the analysis continues. When compounded with uncertainty in later stages of analysis, even small uncertainties in monitored ozone levels can lead to large impacts on total benefits.

Given the wide variety of sources for uncertainty and the potentially large degree of uncertainty about any specific estimate, we characterized uncertainty in two ways, using both a limited scope Monte Carlo analysis and sensitivity analyses.

More than one source of uncertainty usually exists, even for individual end points. This makes it difficult to provide an overall quantified uncertainty estimate, for either individual end points or total benefits. For example, the health impact function used to estimate avoided premature deaths has an associated standard error that represents the statistical error around the effect estimate in the underlying epidemiologic study. In our results, we report a CI based on this standard error, reflecting the uncertainty in the estimated change in incidence of avoided premature deaths. However, this CI omits the contribution of air quality changes, baseline incidence rates, populations exposed, and transferability of the effect estimate to diverse locations. As a result, the reported CI gives a potentially misleading picture about the overall uncertainty in the estimates. This information should be interpreted within the context of the larger uncertainty surrounding the entire analysis.

We used Monte Carlo methods to generate CIs around the estimated health impact and dollar benefits. Monte Carlo simulation uses random sampling from distributions of parameters to characterize the effects of uncertainty on output variables, such as incidence of premature mortality. Distributions for individual effect estimates are based on the reported standard errors in the epidemiologic studies. Distributions for unit values are described in Table 4.

## Results and Implications

Table 5 summarizes the incidence and valuation for each year associated with two attainment simulation methods, percentage and quadratic. Table 6 provides the results averaged across the 3 years. In addition to the mean incidence and valuation estimates, we have included a 5th and 95th percentile estimate in Table 6, based on the Monte Carlo simulations described above. To calculate the air quality values under each attainment scenario, we rolled back the ozone monitor data so that the fourth highest daily maximum 8-hr average just met the level required to attain the standard. This approach will likely understate the benefits that would occur because of implementation of actual controls to reduce ozone precursor emissions. These controls would likely reduce ozone concentrations at all monitors within a nonattainment area, rather than just at those monitors with out-of-attainment ozone values. Therefore, our results are an underestimate of the likely benefits of attaining the ozone standard. In all of the primary analytical cases, we used VNA with no distance limit and assumed a 40 ppb background level for the attainment metric and an hourly background level of zero.

The results for 2000 and 2001 are similar in magnitude, whereas the results for 2002 are roughly twice that of each of the prior 2 years. The simple average of benefits (including premature mortality) across the 3 years is $5.7 billion (90% CI, 0.6–15.0) for the quadratic rollback simulation method and $4.9 billion (90% CI, 0.5–14.0) for the percentage rollback simulation method. Average benefits without premature mortality are $200 million (90% CI, 72–350) for the quadratic rollback method and $160 million (90% CI, 65–310) for the percentage rollback method. Including premature mortality in our estimates had the largest impact on the overall magnitude of benefits: Premature mortality benefits account for more than 95% of the total benefits we can monetize.

Table 7 shows the impact on incidence of health impacts of a range of assumptions regarding how we rolled back the ozone monitor values. We considered the impact of ordinality—that is, of choosing the first versus the fourth highest daily maximum 8-hr average—and we chose a range of alternative background levels. Regardless of attainment simulation method, ordinality had the largest apparent impact, with roughly a factor of 2–3 separating results between the first highest and fourth highest 8-hr maximum. It is important to note that health impacts are likely to occur whenever the 8-hr daily maximum is elevated, not just when the number of exceedances is greater than four. Although the standard reflects the underlying health science and seeks to protect public health, it does not guarantee zero health impacts. That said, the magnitude of the difference in this analysis is still somewhat surprising.

Two elements contribute to this result. First, certain monitors will meet the standard with an ordinality of four but will not meet the standard with an ordinality of one. That is, some monitors may have one metric value > 84 ppb but will not have four such values. As discussed above, monitors that meet the standard are not adjusted at all, so these monitors will have a large impact on the results. Second, certain monitors have a small number of outlier metric values that are much higher than all of the rest. Because the rollback strategies both adjust all metric values, basing a rollback on these outlier values can cause much higher reductions across the entire year.

The impact of attainment metric background and the hourly background depended on attainment simulation method. Under the percentage rollback attainment simulation method, shifting the attainment metric background from 40 to 0 increased impacts by roughly a factor of 2, but the same shift under the quadratic rollback method had no significant impact on results. However, shifting the hourly background level from 0 to 40 under the quadratic rollback method resulted in a roughly 60% reduction in impacts, while making the same background shift using the percentage rollback method reduced impacts by around a third.

For any particular assumption of background ozone levels, our estimates are likely to understate the actual benefits that would occur from implementing control strategies to attain the 8-hr standard, because of our assumption that only the specific monitors that are out of attainment in any area will realize reductions in ozone levels. Our estimates of benefits in areas of the country with longer ozone seasons, such as California and Texas, will also be underestimates due to our assumption of a fixed ozone season from 1 May to 30 September for the entire nation. Analyses of specific attainment strategies should allow for changes in ambient ozone across all monitors in a nonattainment area, as well as accounting for the variable length of the ozone season. Because there is currently no known threshold for most ozone-related health effects, there is likely to be a significant benefit to reducing ozone concentrations beyond the standard at monitors that currently attain the standard.

Applying a distance limit of 50 km to the VNA method reduced benefits by 3–10%, depending on the year of analysis. Use of a closest monitor algorithm with a 50-km limit reduced benefits by 10–15%, depending on the year of analysis. Most of this difference occurs because approximately 10% of the population lives > 50 km away from an ozone monitor. Detailed sensitivity analyses examining the choice of interpolation method are available on request.

Our estimates of mortality-related benefits of attaining the standards may change, based on emerging meta-analyses of the ozone mortality literature. If these meta-analyses confirm the results of Thurston and Ito (2001), Levy et al. (2001), or the WHO report (2003) meta-analyses, the mean mortality benefits may increase by a factor of 2, suggesting that reductions in premature mortality associated with attainment of the ozone standards might be as high as 1,600 premature deaths avoided annually. This increase would substantially increase the economic value of health impacts as well, potentially up to $10 billion. Using the Jaffe et al. (2003) effect estimates for asthma ED visits in the population 5–34 years of age would have increased the estimated number of avoided admissions by approximately 4.5 times. This suggests that the all-ages estimates based on earlier studies may underestimate impacts in younger populations. Details of the sensitivity analyses examining alternative mortality and morbidity effect estimates are available from the authors.

In this analysis we estimated the health benefits of reducing ozone levels in areas with monitored values that exceed the 8-hr ozone standard. The increasing need to understand the public health impacts of air pollution regulations requires the merging of models and data from many disciplines. Although necessary, this type of multidisciplinary methodology is challenging in complexity and scope. Our approach illustrates the integration of models and data and highlights uncertainties inherent in the end results. The result suggests there may be significant health benefits arising from actions that reduce ozone concentrations in nonattainment areas.

The results of our analysis suggest that moving from current monitored ozone levels to full attainment of the 8-hr standard may yield substantial health benefits. We estimate total benefits (including premature mortality) of meeting the standard as reaching up to $5.7 billion (averaged over 3 years, 2000–2002). These dollar benefits are associated with average annual reductions in health effects, including > 800 avoided premature deaths, > 4,000 avoided hospital admissions, approximately 500 avoided asthma ED visits per year, > 1 million avoided restricted activity days, and > 900,000 avoided school absences.

We provide sensitivity analyses to examine key modeling assumptions. In addition, we could not quantify other uncertainties, such as the importance of unquantified effects and uncertainties in the interpolation of ambient air quality. Inherent in any analysis of health impacts are uncertainties in affected populations, health baselines, incomes, effect estimates, and other factors. The assumptions used to capture these elements are reasonable based on the available evidence. However, these data limitations prevent a full-scale quantitative estimate of the uncertainty associated with estimates of total economic benefits. If one is mindful of these limitations, the magnitude of the benefit estimates presented here can be useful information in expanding the understanding of the public health impacts of attaining the 8-hr ozone standard.

## Figures and Tables

**Table 1:** Distribution of fourth highest maximum daily average O_3_ values across monitors.

	Monitors with value in range (%)		
Range of O_3_ values (ppb)	2000 (1,089 monitors)	2001 (1,120 monitors)	2002 (1,146 monitors)
≤84 (in attainment)	64	61	44
84–89.9	17	18	15
90–99.9	15	16	27
100–109.9	3	4	11
> 110	1	1	3

**Table 2:** Ozone-related health end points included in primary and sensitivity analyses.

Health effect	Applied ages (years)	Description	Ozone metric
Premature mortality	All	Pooled estimate	
		Ito and Thurston (1996)	1-hr daily maximum
		Moolgavkar et al. (1995b)	24-hr daily average
		Samet et al. (1997)	24-hr daily average
	All	Sensitivity	
		WHO (2003)	8-hr average
Respiratory hospital admissions	≥65	Pooled estimate	
		Schwartz (1995): ICD-9 460–519 (all respiratory disease)	24-hr daily average
		Schwartz (1994a, 1994b): ICD-9 480–486 (pneumonia)	
		Moolgavkar et al. (1997): ICD-9 480–487 (pneumonia)	
		Schwartz (1994b): ICD-9 491–492, 494–496 (COPD)	
		Moolgavkar et al. (1997): ICD-9 490–496 (COPD)	
	0 to < 2	Burnett et al. (2001)	24-hr daily average
Asthma-related ED visits	All	Pooled estimate	
		Weisel et al. (1995)	5-hr daily average
		Cody et al. (1992)	5-hr daily average
		Stieb et al. (1996)	24-hr daily average
	5–34	Sensitivity	
		Jaffe et al. (2003)	8-hr daily maximum
Other health effects School loss days*^a^*		Pooled estimate	
	5–17	Gilliland et al. (2001)	8-hr daily average
	5–17	Chen et al. (2000)	1-hr daily maximum
MRADs	18–65	Ostro and Rothschild (1989)	24-hr daily average

COPD, chronic obstructive pulmonary disease.

a Gilliland et al. (2001) studied children 9 and 10 years of age. Chen et al. (2000) studied children 6–11 years of age. Based on recent advice from the National Research Council (2002) and the U.S. EPA Science Advisory Board Health Effects Subcommittee, we have calculated reductions in school absences for all school-age children based on the biologic similarity among children 5–17 years of age.

**Table 4:** Unit values for economic valuation of health end points (2000 US$).

Health end point	Description	Mean estimate adjusted for income growth to 2000*^a^*	Distribution
Mortality	VSL based on 26 studies	$6.5 million per statistical life	The $6.5 million estimate is the mean of a Weibull distribution fitted to the estimates from 26 value-of-life studies identified in U.S. EPA section 812 reports (e.g., U.S. EPA 1999) as “applicable to policy analysis.” Five of the 26 studies are contingent valuation studies, which directly solicit WTP information from surveyed subjects. The remainder are wage-risk studies, which base WTP estimates on estimates of the additional compensation demanded for riskier jobs.
Hospital admissions	All respiratory, ≥65 years of age	$18,353 per admission	No distributions available. The COI point estimates (lost earnings plus direct medical costs) are based on ICD-9 code-level information (e.g., average hospital care costs, average length of hospital stay, and weighted share of total COPD category illnesses) reported in Agency for Healthcare Research and Quality (2000).
	All respiratory, 0 to < 2 years of age	$7,741 per admission	
ED visits	Asthma-related	$286 per visit	No distribution available. The COI point estimate is the simple average of two unit COI values: $312 from Smith et al. (1997), and $261 from Stanford et al. (1999).
Minor effects	MRAD	$52 per day	Median WTP estimate to avoid one MRAD from Tolley et al. (1986). Distribution is assumed to be triangular with a minimum of $22 and a maximum of $83. Range is based on assumption that value should exceed WTP for a single mild symptom (the highest estimate for a single symptom —for eye irritation—is $16.00) and be less than that for a work loss day. The triangular distribution acknowledges that the actual value is likely to be closer to the point estimate than either extreme.
School absences		$75 per day	No distribution available.

a The derivation of each of the estimates is discussed in the text. COI-based unit values are not adjusted for income growth because they are based on current costs and wage rates. These include hospital admissions, ED visits, and school absences.

**Table 3:** National average baseline incidence rates.

			Rate per 100 people per year by age group (years)*^b^*						
End point	Source*^a^*	Notes	< 18	18–24	25–34	35–44	45–54	55–64	≥ 65
Mortality	CDC compressed mortality file, nonaccidental, accessed through CDC WONDER (1996–1998)	Nonaccidental	0.025	0.022	0.057	0.150	0.383	1.006	4.937
Respiratory hospital admissions	1999 NHDS public use data files	Incidence	0.043	0.084	0.206	0.678	1.926	4.389	11.629
Asthma ED visits	2000 NHAMCS public use data files 1999 NHDS public use data files	Incidence	1.011	1.087	0.751	0.438	0.352	0.425	0.232
MRADs	Ostro and Rothschild (1989)	Incidence	NA	780	780	780	780	780	NA
School loss days	U.S. Department of Education (1996) and 1996 HIS (Adams et al. 1999, Table 47), estimate of 180 school days per year	All-cause	990.0	NA	NA	NA	NA	NA	NA

Abbreviations: CDC, Centers for Disease Control and Prevention; NA, not applicable.

a The following abbreviations are used to describe the national surveys conducted by the National Center for Health Statistics: CDC WONDER, CDC Wide-Ranging Online Data for Epidemiological Research (CDC 2004a); HIS, National Health Interview Survey (CDC 2004b); NHDS, National Hospital Discharge Survey (CDC 2004c); NHAMCS, National Hospital Ambulatory Medical Care Survey (CDC 2004d).

b All of the rates reported here are population-weighted incidence rates per 100 people per year. Additional details on the incidence and prevalence rates, as well as the sources for these rates are available upon request.

**Table 5:** Summary of estimated annual health benefits of attaining the 8-hr standard.

	2000		2001		2002	
End point	Cases	Economic value*^a^*	Cases	Economic value*^a^*	Cases	Economic value*^a^*
Quadratic rollback						
Premature mortality	560	3,600	670	4,400	1,300	8,400
Hospital admissions, respiratory, adults	1,500	27	1,900	34	3,600	67
Total hospital admissions, respiratory, children	1,700	13	1,600	13	2,900	23
ED visits for asthma	370	0.11	410	0.12	750	0.22
School absences	740,000	55	780,000	59	1,400,000	110
MRADs	950,000	49	1,100,000	55	2,000,000	100
Total economic value of health changes						
With premature mortality		3,700		4,600		8,700
Without premature mortality		140		160		300
Percentage rollback						
Premature mortality	500	3,200	590	3,300	1,160	7,600
Hospital admissions, respiratory, adults	1,300	24	1,600	17	3,200	60
Total hospital admissions respiratory, children	1,500	12	1,500	3	2,700	21
ED visits for asthma	330	0.10	360	0.05	680	0.20
School absences	660,000	50	700,000	27	1,300,000	97
MRADs	850,000	44	950,000	18	1,800,000	93
Total economic value of health changes						
With premature mortality		3,300		3,400		7,900
Without premature mortality		130		70		270

a Million (2000 US$).

**Table 6:** Estimated average annual health benefits of attaining 8-hr standard (2000–2002 monitor data).

		Cases			Economic value (million 2000 US$)		
Endpoint	Age range (years)	5th	Mean	95th	5th	Mean	95th
Quadratic rollback							
Premature mortality	All	290	840	1,600	500	5,500	15,000
Hospital admissions, respiratory, adults	≥65	530	2,300	4,600	10	43	84
Total hospital admissions, respiratory, children	0 to <2	1,100	2,100	3,100	8.70	16	24
ED visits for asthma	All	180	510	870	0.05	0.15	0.26
School absences	5–17	350,000	970,000	1,700,000	26	75	130
MRADs	18–64	670,000	1,400,000	2,000,000	28	68	110
Total economic value of health changes							
With premature mortality					570	5,700	15,000
Without premature mortality					70	200	350
Percentage rollback							
Premature mortality	All	260	750	1,400	470	4,700	13,000
Hospital admissions, respiratory, adults	≥65	470	2,000	4,100	8.70	34	76
Total hospital admissions, respiratory, children	0 to <2	970	1,900	2,800	7.70	12	22
ED visits for asthma	All	150	460	770	0.04	0.12	0.23
School loss days	5–17	310,000	890,000	1,500,000	23	58	120
MRADs	18–64	610,000	1,200,000	1,800,000	26	52	110
Total economic value of health changes							
With premature mortality					530	4,900	14,000
Without premature mortality					65	160	310

5th and 95th percentile estimates based on the Monte Carlo simulations described in the text.

**Table 7:** Sensitivity of mean estimated annual health effects of attaining the 8-hr standard relative to 2001 monitor values, to ordinality, attainment metric background (AMB), and hourly background (HB) (cases).

End point	Base	A	B	C	D
Quadratic rollback					
Premature mortality	700	1,600	700	300	500
Hospital admissions, respiratory					
Adults	1,900	4,600	2,000	700	1,300
Children	1,630	3,890	1,730	1,110	2,010
ED visits for asthma	410	970	430	220	400
School loss days	780,000	1,900,000	840,000	520,000	950,000
MRADs	1,100,000	2,600,000	1,100,000	430,000	760,000
Percentage rollback					
Premature mortality	600	2,800	1,100	400	900
Hospital admissions, respiratory					
Adults	1,600	8,100	3,100	1,100	2,500
Children	1,460	6,840	2,620	1,770	4,010
ED visits for asthma	360	1,900	650	340	750
School loss days	700,000	3,300,000	1,300,000	840,000	1,900,000
MRADs	950,000	4,500,000	1,700,000	660,000	1,400,000

Sensitivity tests (Base, A–D) were conducted using the VNA interpolation method with no distance limit. Ordinality refers to the *n*th highest value used to determine attainment with the level of the standard. For example, the form of the 8-hr standard specifies the fourth highest maximum 8-hr average. The ordinality in this case is 4. Attainment metric background (AMB) refers to the assumed level of the attainment standard (fourth highest maximum 8-hr average) that would exist in the absence of domestic man-made emissions of ozone precursors. Hourly background (HB) refers to the assumed level of ozone at any hour that would exist in the absence of domestic man-made emissions of ozone precursors. Ordinality, AMB, and HB are, respectively, 4, 40, 0 for Base; 1, 0, 0 for A; 4, 0, 0 for B; 4, 40, 40 for C; and 1, 40, 40 for D.
